# Transthoracic echocardiographic reference values of the aortic root: results from the Hamburg City Health Study

**DOI:** 10.1007/s10554-021-02354-5

**Published:** 2021-07-29

**Authors:** Jan-Per Wenzel, Elina Petersen, Julius Nikorowitsch, Juliana Senftinger, Christoph Sinning, Matthias Theissen, Johannes Petersen, Hermann Reichenspurner, Evaldas Girdauskas

**Affiliations:** 1 Department of Cardiology, University Heart and Vascular Centre Hamburg, Hamburg, Germany; 2 German Centre for Cardiovascular Research (DZHK), Partner Site Hamburg/Kiel/Luebeck, Hamburg, Germany; 3 Department of Cardiovascular Surgery, University Heart Centre Hamburg, Hamburg, Germany; 4 Epidemiological Study Centre, Hamburg, Germany

**Keywords:** Aortic root, Echocardiography, Reference values, HCHS, Hamburg City Health Study, Thoracic aorta

## Abstract

Here we generate up-to-date reference values of transthoracic echocardiographic aortic root dimensions matched by sex, age, and body surface area (BSA) derived from the population-based Hamburg City Health Study (HCHS) cohort. In 1687 healthy subjects (mean age 57.1 ± 7.7, 681 male and 1006 female), derived from the first prospectively-recruited 10,000 HCHS participants, dimensions of the aortic root were measured in systole and diastole using state-of-the-art 2-dimensional transthoracic echocardiography. Diameters were assessed at four levels: aortic annulus, Sinus of Valsalva, sinotubular junction, and ascending aorta. Female sex was associated with significantly smaller absolute aortic root dimensions, while indexing for BSA resulted in a reverse effect at all levels. There was a strong age dependency of all aortic root diameters as well as aortic annulus/sinotubular junction ratio for both sexes. Multivariate analysis revealed age, sex, weight, height, and BSA to be significant determinants of aortic root size. Finally, formulas were generated for the calculation of individual aortic root reference values considering age, sex, weight, and height. We provide population-based reference values of aortic root diameters based on a standardized transthoracic echocardiographic protocol of the population-based HCHS which may support the diagnosis, monitoring, and treatment of aortic root disease.

**Supplementary Information:**

The online version contains supplementary material available at 10.1007/s10554-021-02354-5.

## Introduction

Aortic root dilatation is a common and multifactorial condition influenced by genetics, hemodynamic-rheological factors, and comorbidities [1]. If significant and progressive, it may lead to aortic valve regurgitation (AR) [2, 3]. Furthermore, an increased aortic root diameter has been shown to be an independent predictor of acute aortic syndrome which is associated with an increased morbidity and mortality [3–5]. Transthoracic echocardiography (TTE) is the most commonly used imaging modality to evaluate the aortic root in the daily clinical practice. This is predominantly due to its wide availability, non-invasiveness, ease of use, and reproducibility. Echocardiographic screening, grading, and surveillance of aortic root aneurysms has a major impact on the patient care. However, the assessment of the aortic root is highly dependent on the acoustic window, proper alignment, the exact location of measurements as well as the availability of validated reference values. Indeed, most of the previously published aortic root reference studies are potentially biased by limited sample sizes, heterogenous measuring techniques and selective study populations [6–9].

The Hamburg City Health Study (HCHS, www.hchs.hamburg) is a single-centre, prospective, long-term, population-based cohort study which targets the interaction of socioeconomic risk factors, modern imaging techniques, physiological measurements, and clinical variables [10]. HCHS will finally include a random sample of approximately 45,000 inhabitants from the general population of Hamburg, Germany aged 45 to 74 years. Based on the inclusion of a sample of the first 10,000 HCHS study participants, the present study aims to define 2-dimensional TTE aortic root reference values derived from a standardized echocardiographic protocol adjusted for age, sex, height, weight, and BSA.

## Methods

### Study population

**Fig. 1:**
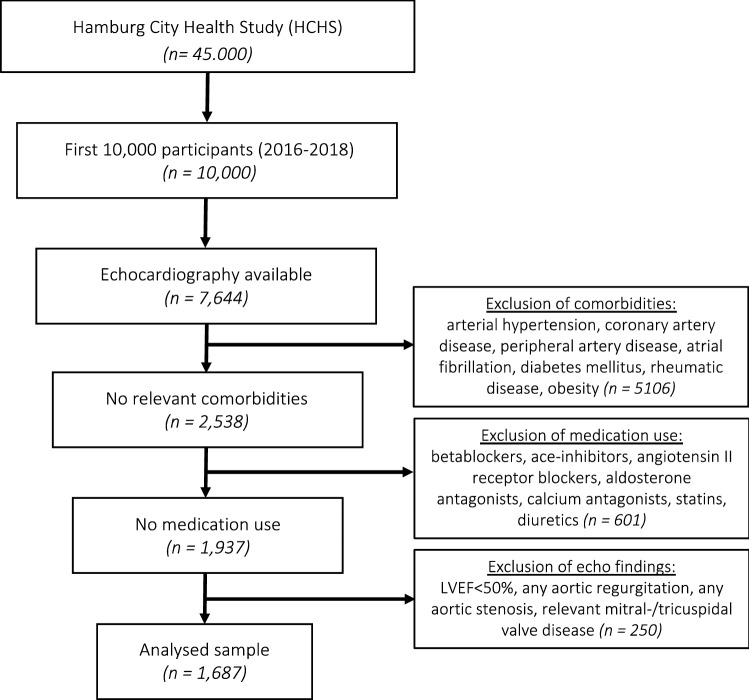
Study PRISMA. From a total of 7644 subjects with available TTE examination and aortic root measurements, 5106 were excluded due to comorbidities, 601 subjects were excluded due to medication use, and 250 due to pathologic echocardiographic findings. Consequently, 1687 subjects were considered applicable for the measurement and calculation of aortic root reference values matched by age, sex, and body surface area. *LVEF* left ventricular ejection fraction

Our study population included a sample of the first 10,000 HCHS participants who received transthoracic ultrasound evaluation. Of 10,000 study individuals, 7644 received TTE with evaluation of the aortic root. Of those 7644 individuals, we excluded 5957 individuals due to arterial hypertension (blood pressure at rest > 140/90 mmHg), medication (betablockers, ace-inhibitors, angiotensin II receptor blockers, aldosterone antagonists, statins, diuretics), coronary artery disease, peripheral artery disease, atrial fibrillation, diabetes mellitus, rheumatic disease, obesity (BMI > 35 kg/m^2^), moderate/severe aortic regurgitation, any aortic stenosis, relevant mitral-/tricuspid valve disease, bicuspid aortic valve, left ventricular ejection fraction (LVEF) below 50% or insufficient image quality to perform standardised measurements. Hence, 1,687 individuals served as our study population for the current analysis (Fig. 1).

All measurements were conducted between 2016 and 2018 during a one-day baseline visit at the HCHS Epidemiological Study Centre Hamburg-Eppendorf, Germany, according to the published study protocol [10]. The research protocol was approved by the HCHS steering board and the local ethics committee (PV5131, Medical Association Hamburg). All participants gave written informed consent.

### TTE image acquisition and analysis

TTE examinations were systematically performed on dedicated ultrasound machines (Siemens Acuson SC2000 Prime, Siemens Healthineers, Erlangen, Germany) using grey-scale second-harmonic imaging technique at the HCHS Epidemiological Study Centre Hamburg-Eppendorf, Germany. The TTE examination followed a strictly standardized protocol which included all the established standard echocardiographic views as well as Doppler velocimetry and 3D visualization. All loops were recorded in native DICOM format and included three ECG-triggered heartbeats. For the assessment of the aortic root, the transthoracic parasternal long axis view of the left ventricle (LV) with focused zoom of the left ventricular outflow tract (LVOT) and the aortic root was recorded at breath hold in consent with the current ASE/EACVI guidelines [11]. To improve image quality, manual adjustments of image depth, sector width, frequency, and gain intensity were applied. For qualitative and quantitative image analysis standard operating procedures (SOP) were defined in agreement with the current guidelines of the American Society of Echocardiography (ASE) and the European Association of cardiovascular imaging (EACVI) [11, 12]. All TTE studies were independently quantified three times by three different investigators at a single reading centre, all blinded to the clinical information. For the assessment of intra-observer reproducibility, a set of 80 TTE studies was analysed twice by each investigator. The commercially available and established Siemens syngo SC2000 software was used (Siemens syngo SC 2000 Version 4.0, Siemens Healthineers, Erlangen, Germany) for echocardiography data analysis.

**Fig. 2:**
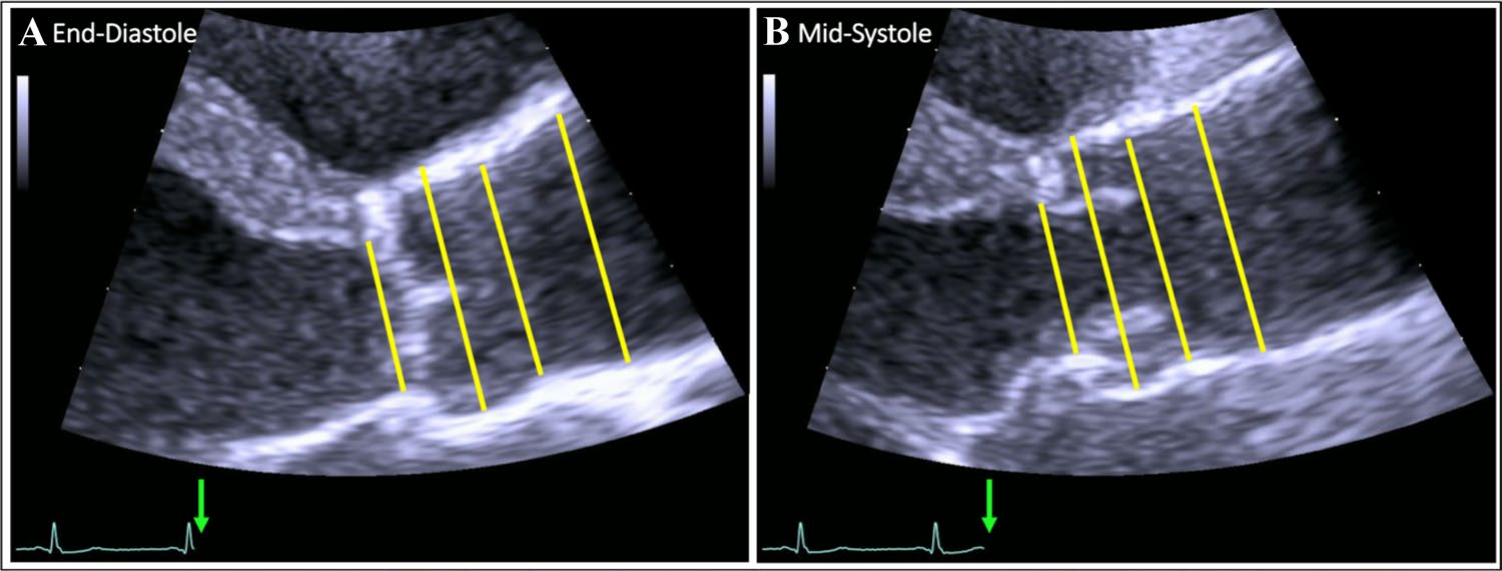
Echocardiographic measurements of the aortic root in end-diastole (**A**) and mid-systole (**B**). From left to right: (1) aortic annulus, (2) sinus of Valsalva, (3) sinotubular junction, and (4) proximal ascending aorta within 2 cm range of the STJ. The green arrows mark the measurement time points in relation to the ECG

Systematic measurements of the aortic root were performed perpendicular to the proximal aortic axis in end-diastole (ED), (i.e., immediately before aortic valve opening) as well as in mid-systole (MS) including the following: (a) aortic annulus (AoAn), (b) sinus of Valsalva (SoV), (c) sinotubular junction (STJ), and (d) proximal ascending aorta (AscAo) at 2 cm range from the STJ as published before by our group [13]. AoAn was measured as the largest diameter between the hinge points of the non- and right coronary cusps of aortic valve, using the inner-edge to inner-edge (II) convention. According to the HCHS consensus, the SoV was measured using the leading-edge to leading-edge (LL) convention, while II convention was used for the measurements of the STJ and AscAo (Fig. 2).

Left sided volumes and left ventricular ejection fraction were calculated using the two-dimensional biplane method of disks summation (modified Simpson’s rule). In case of insufficient image quality for tracing the blood–tissue-border, no measurements were conducted. Left-sided diameters were measured in parasternal long-axis view (2-dimensional). Valvular heart disease was detected by a combination of visual assessment, colour Doppler, and continuous wave-Doppler following the current ASE and EACVI guidelines [14]. Aortic valve cuspidity was visually assessed in parasternal short- and long-axis view.

### Statistical analysis

Magnitudes of continuous variables were presented as mean ± standard deviation (SD) or median ± interquartile range (IQR), accordingly. Intra-class correlation coefficient (ICC) estimates and their 95% confident intervals (CI) were calculated based on a mean-rating, consistency, 2-way mixed-effects model [15].

The unpaired t-test was used to analyse differences between groups. For non-normally distributed variables, the Mann–Whitney U test was used instead. Pearson’s correlation coefficient was used to quantify correlation between end-diastolic and mid-systolic measurements. For multiple group comparisons, overall significance levels were obtained using one-way anova. For multiple pairwise comparison against the base-mean, the t-test was used.

Multivariable linear regression was used to assess the association between aortic root diameters as dependent variables and either weight and height or BSA as independent variables. Weight, height, and BSA were not included in the same model to avoid multicollinearity. Age and sex were included as covariates in all regression models. Collinearity was controlled for by determining the variance inflation factor (VIF) for linear regression models. Inclusion of other baseline characteristics did not improve the model fit. Differences were considered statistically significant at a two-sided p-value level of 0.05 after post-hoc correction using the Bonferroni-Holm method. All statistical analyses were performed using R (version 4.0.3). A list of the used packages and versions can be found in the appendix.

## Results

### Baseline characteristics

A total of 1687 subjects (681 male and 1006 female) from the first 10,000 HCHS participants with a mean age of 57.1 ± 7.7 (range 46–78) was included in the study. Per protocol, subjects were healthy adults with normal anthropometric and clinical characteristics (Table 1). Men showed higher BMI, weight, height, BSA, blood pressure, and left ventricular mass as well as larger left sided cavities as compared to the women. In contrast, women had higher LVEF.

**Table 1:** Characteristics of the study population

	Male	Female	p-value
	(n = 681)	(n = 1006)	
Antrophometrics			
Age	58.3 (8.0)	57.9 (7.4)	0.310
Weight (kg)	82.6 (11.5)	67.4 (10.8)	< 0.001
Height (cm)	179.7 (6.9)	166.3 (6.6)	< 0.001
BSA (m^2^)	2.0 (0.2)	1.7 (0.1)	< 0.001
BMI (kg/m^2^)	25.5 (3.0)	24.4 (3.6)	< 0.001
Waist circumference, cm	95.0 (9.5)	84.6 (10.3)	< 0.001
Heart rate, bpm	65.9 (10.6)	68.9 (8.9)	< 0.001
Systolic bp (mmHg)	128.4 (9.5)	123.7 (11.2)	< 0.001
Diastolic bp (mmHg)	78.9 (6.6)	76.7 (7.3)	< 0.001
Laboratories			
Hemoglobin, g/dl	14.9 (0.9)	13.6 (0.9)	< 0.001
LDL (mg/dl)	122.6 (33.3)	123.6 (35.0)	0.561
GFR (ml/min)	87.5 (11.7)	87.0 (12.7)	0.454
NT-proBNP (ng/l)	67.8 (113.2)	102.7 (88.8)	< 0.001
hsCRP, mg/l	0.2 (0.4)	0.2 (0.3)	0.977
Fasting glucose (mg/dl)	91.8 (9.5)	87.8 (8.3)	< 0.001
Echocardiographic data			
LVEF (%)	*58.6 (4.0)*	*60.2 (4.5)*	< *0.001*
LVEDV (ml/m^2^)	*130.9 (29.6)*	*103.7 (23.2)*	< *0.001*
LVESV (ml)	*54.2 (13.2)*	*41.2 (10.8)*	< *0.001*
LVEDD (mm)	*49.6 (4.5)*	*45.6 (4.1)*	< *0.001*
LV mass indexed (g)	*85.8 (17.2)*	*73.6 (13.9)*	< *0.001*
LAVI, ml/m^2^	*27.5 (7.4)*	*25.9 (7.1)*	< *0.001*
E/e′ ratio	*6.6 (1.5)*	*7.2 (1.8)*	< *0.001*
TAPSE	*25.4 (4.3)*	*24.8 (4.4)*	*0.007*
TR V_max_	*2.3 (0.2)*	*2.3 (0.2)*	*0.409*

Continuous variables are presented as mean and standard deviation, and categorical variables are presented as absolute numbers and percentages

*BMI* body mass index,* bp* blood pressure,* BSA* body surface area,* bp* blood pressure,* LVEF* left ventricular ejection fraction,* LVEDV* left ventricular end-diastolic volume, *LVESV* left ventricular end-systolic volume, *LVEDD* left ventricular end-diastolic diameter,* sd* standard deviation

p-value for intergroup differences

### Feasibility and reliability of measurements

AoAn MS and SoV ED showed the highest feasibility with approximately 97%, while AscAo MS was assessable in only 51.5% of our cohort. There were no sex-specific differences in feasibility (Supplements, Table 6). Interobserver reproducibility values were high for all variables, as shown by ICC ranging from 0.95 to 0.99. Intra-observer reproducibility was similar, with ICCs ranging between 0.90 and 0.99.

### Aortic root reference values

**Fig. 3:**
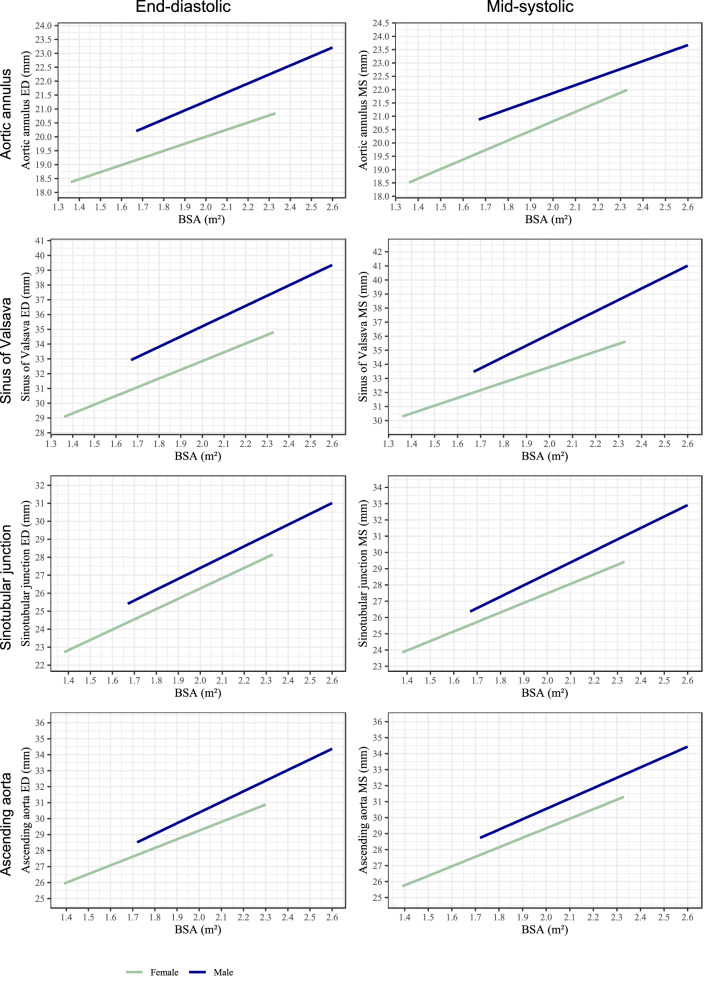
Nomograms of aortic root diameters for women and men ≤ 60 years at end-diastole and mid-systole in relation to body surface area. X-axis represents body surface area in square meters, y-axis represents aortic root diameters in millimeters. *BSA* body surface area

**Fig. 4:**
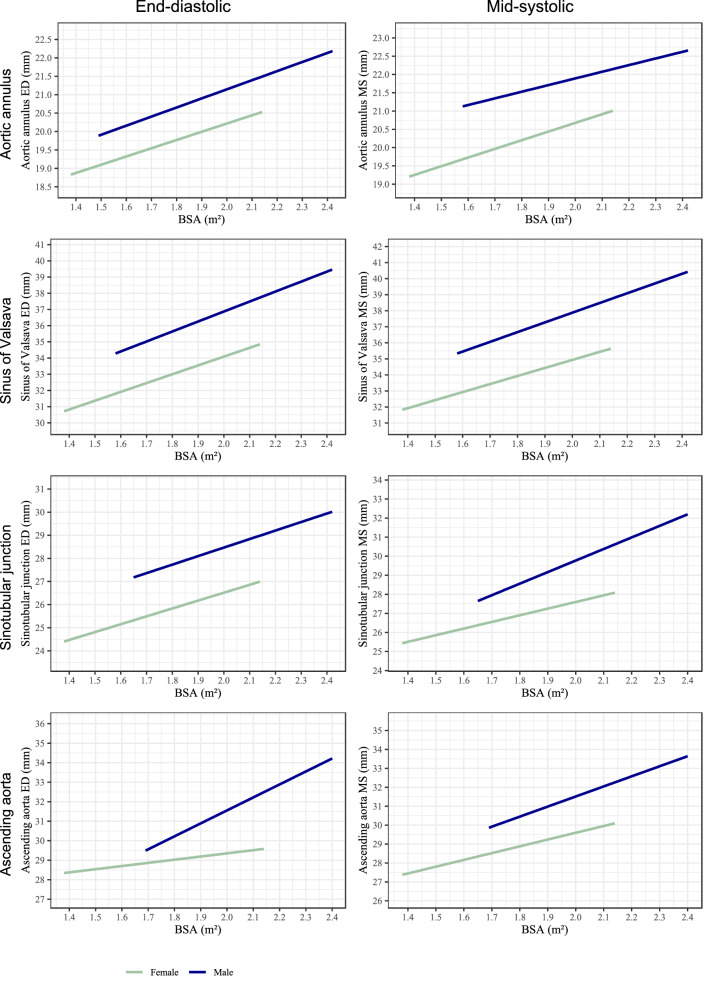
Nomograms of aortic root diameters for women and men > 60 years at end-diastole and mid-systole in relation to BSA. X-axis represents BSA in square meters, y-axis represents aortic root diameters in millimeters. *BSA* body surface area

Absolute aortic root diameters were larger at all four measurement levels in men as compared to women, both in end-diastole and mid-systole. Indexing of aortic root diameters to BSA had a reverse effect and revealed significantly larger aortic root diameters for women (Table 2). Both non-indexed and indexed aortic root diameters increased significantly with increasing age in males and females (Supplement Table 5). Multiple linear regression analysis showed significant correlations between age, sex, BSA, weight, height, and aortic root diameter (Table 3). Integrating further variables, e.g. blood pressure (Supplement Table 7), waist circumference, left ventricular end-diastolic diameter, and left ventricular end-diastolic volume, did not relevantly improve the predictive capacity of our model. The strongest correlation in the multivariate model was found for the prediction of SoV diameters (R^2^ = 0.39 (BSA model); R^2^ = 0.39 (height model)). Age showed the weakest association with AoAn diameter in both models (i.e., ß = 0.010; p ≤ 0.001 in the BSA model and ß = 0.011; p ≤ 0.001 in the height model). For visualization purpose, we prepared age-, sex-, height- and BSA-adjusted nomograms for all measured aortic root diameters (Figs. 3, 4, Supplements Fig. 6).

**Table 2:** Echocardiographic measurements of the aortic root—absolute and indexed to BSA

	Absolute diameters (mm)		Diameters indexed to BSA (mm/m^2^)			
	Male	Female	p-value	Male	Female	p-value
	(n = 681)	(n = 1006)		(n = 681)	(n = 1006)	
ED aortic annulus	21.97 [21.86–22.08]	19.97 [19.89–20.05]	< 0.001	10.92 [10.84–10.99]	11.48 [11.42–11.55]	< 0.001
ED sinus of valsalva	21.27 [21.15–21.39]	19.45 [19.37–19.54]	< 0.001	10.61 [10.53–10.68]	11.19 [11.12–11.26]	< 0.001
ED sinotubular junction	35.96 [35.7–36.21]	31.83 [31.65–32.01]	< 0.001	17.88 [17.73–18.03]	18.28 [18.16–18.41]	< 0.001
ED ascending aorta	27.91 [27.68–28.13]	25.15 [24.97–25.32]	< 0.001	13.88 [13.76–14.01]	14.42 [14.3–14.53]	< 0.001
MS aortic annulus	30.95 [30.58–31.31]	28.31 [28.04–28.58]	< 0.001	15.37 [15.18–15.57]	16.19 [16.01–16.38]	< 0.001
MS sinus of valsalva	31.05 [30.71–31.39]	28.17 [27.92–28.42]	< 0.001	15.42 [15.23–15.6]	16.12 [15.96–16.29]	< 0.001
MS sinotubular junction	36.95 [36.67–37.24]	32.86 [32.66–33.05]	< 0.001	18.38 [18.22–18.54]	18.86 [18.72–19]	< 0.001
MS ascending aorta	29.18 [28.89–29.46]	26.29 [26.09–26.48]	< 0.001	14.52 [14.37–14.68]	15.05 [14.93–15.18]	< 0.001

Continuous variables are presented as mean and 95% confidence interval. Abbreviations as in Table 1

p-value for intergroup differences

**Table 3:** Multiple linear regression analyses of absolute aortic root diameters (mm) measured in end-diastole with BSA or height/weight as independent variables adjusted for age and sex

End-diastolic diameters	BSA model			Height model		
	R^2^	ß	p-value	R^2^	ß	p-value
Aortic annulus	0.35			0.36		
Age		0.010	0.730		0.011	0.526
Male sex		1.088	< 0.001		1.075	< 0.001
BSA		2.717	< 0.001			
Height					0.025	0.002
Weight					0.027	< 0.001
Sinus of valsalva	0.39			0.39		
Age		0.099	< 0.001		0.106	< 0.001
Male sex		2.390	< 0.001		2.160	< 0.001
BSA		6.327	< 0.001			
Height					0.087	< 0.001
Weight					0.051	< 0.001
Sinotubular junction	0.30			0.30		
Age		0.072	< 0.001		0.079	< 0.001
Male sex		1.335	< 0.001		1.103	< 0.001
BSA		5.358	< 0.001			
Height					0.080	< 0.001
Weight					0.040	< 0.001
Ascending aorta	0.19			0.19		
Age		0.072	< 0.001		0.071	< 0.001
Male sex		1.215	0.002		1.308	0.002
BSA		5.467	< 0.001			
Height					0.031	< 0.001
Weight					0.064	< 0.001

*BSA* body surface area

**Fig. 5:**
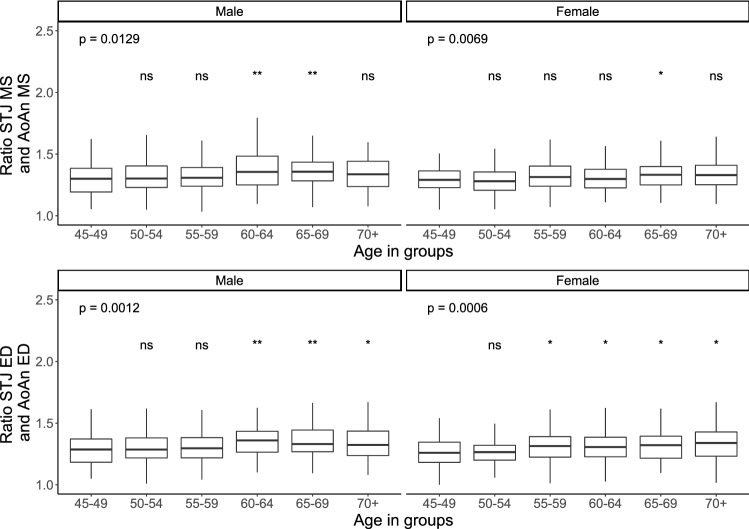
Ratio of sinotubular junction and aortic annulus plotted against age categories stratified by sex. P value for overall difference between age groups. Significance levels for pairwise comparison against the age group 45–49. ns = p > 0.05, *p ≤  0.05, **p ≤  0.01, ***p ≤ 0.001, ****p ≤  0.0001. *AoAn* aortic annulus,* ED* end-diastolic,* MS* mid-systolic, *STJ* sinotubular junction

For all aortic root diameters, end-diastolic measurements correlated strongly with mid-systolic measurements in men and in women (AoAn: women: R = 0.63, p < 0.0001, men: R = 0.70, p < 0.0001; SoV: women: R = 0.91, p < 0.0001, men: R = 0.92, p < 0.0001; STJ: women: R = 0.87, p < 0.0001, men: R = 0.85, p < 0.0001; AscAo: women: R = 0.70, p < 0.0001, men: R = 0.76, p < 0.0001). The STJ/AoAn ratio increased significantly with an advancing age in men and in women, a finding which highlights a stronger age-dependency of STJ growth as compared to the AoAn (Fig. 5).

In line with this finding, our multiple linear regression analysis showed a stronger association between STJ diameter and age as compared to the correlation between age and AoAn diameter (Table 3). Based on our multiple linear regression analysis, formulas for the calculation of individual reference values of normal aortic root diameters were prepared which integrate age, sex, height, and weight (Table 4).

**Table 4:** Formulas for specific calculation of individual end-diastolic, absolute aortic root reference values derived from multiple linear regression analyses

Aortic measurement		Formula for calculation of normal aortic root diameter
Aortic annulus	Lower	AoAn = 10.828 + Age (years) * 0.001 + 0 (Females) | 1 (Males) * 0.871 + Height (cm) * 0.013 + Weight (kg) * 0.020
	Higher	AoAn = 14.970 + Age (years) * 0.020 + 0 (Females) | 1 (Males) * 1.278 + Height (cm) * 0.037 + Weight (kg) * 0.034
Sinus of Valsalva	Lower	SoV = 3.483 + Age (years) * 0.086 + 0 (Females) | 1 (Males) * 1.731 + Height (cm) * 0.062 + Weight (kg) * 0.036
	Higher	SoV = 12.129 + Age (years) * 0.125 + 0 (Females) | 1 (Males) * 2.589 + Height (cm) * 0.113 + Weight (kg) * 0.065
Sinotubular junction	Lower	STJ = 0.600 + Age (years) * 0.061 + 0 (Females) | 1 (Males) * 0.707 + Height (cm) * 0.056 + Weight (kg) * 0.026
	Higher	STJ = 8.562 + Age (years) * 0.097 + 0 (Females) | 1 (Males) * 1.499 + Height (cm) * 0.103 + Weight (kg) * 0.054
Ascending aorta	Lower	AscAo = 8.189 + Age (years) * 0.041 + 0 (Females) | 1 (Males) * 0.655 + Height (cm) * -0.007 + Weight (kg) * 0.040
	Higher	AscAo = 21.214 + Age (years) * 0.101 + 0 (Females) | 1 (Males) * 1.961 + Height (cm) * 0.069 + Weight (kg) * 0.087

Models integrate age, sex, height, and weight. Imputation of values of a specific person leads to prediction of aortic root diameters

## Discussion

The present study provides standardized echocardiographic aortic root reference values derived from the single-centre and population-based HCHS study based on state-of-the-art cardiac ultrasound.

### Impact of the methodological approach

Most of the published echocardiographic studies on aortic root dimensions used primarily M-mode technique providing high spatial resolution at the cost of angle dependency [6, 7]. Nevertheless, M-mode echocardiography was superseded and replaced by 2D-technique. In our current study we used a novel approach to define specific aortic root diameters in 2D-echocardiography using a combination of two measuring modalities: the LL and II convention. While the LL convention is the most used measuring technique for echocardiography in adults, other imaging modalities (i.e., CMR and CT) utilize predominantly the II convention. In recent ASE/EACVI guidelines, the consensus has been obtained to continue using the LL convention for echocardiography due to reduced artefacts with LL measurements and insufficient validation of the II method [11, 12]. Nevertheless, the LL convention has important limitations: For AoAn measurements, the detection of the leading edge is severely limited by aortic valve degeneration, especially in the setting of valvular calcification. Therefore, the ASE/EACVI recommends measuring the AoAn using the II convention. The STJ has a much thinner wall and more extensive movement amplitude during the cardiac cycle as compared to the SoV, which complicates the definition of the anterior leading edge and, therefore, the II measurement method might be advantageous. Furthermore, previous data indicate that the LL convention tends to overestimate aortic diameters as compared to CT measurements which predominantly use the II convention [16]. Therefore, the interdisciplinary consensus in the HCHS study was to measure AoAn, STJ, and AscAo diameters using the II convention, while SoV diameters were defined using the LL convention.

Another important feature of our echocardiographic analysis is the systematic measurement of all aortic root diameters in systole and diastole. Until now, there is no consensus regarding the value and the clinical impact of mid-systolic (MS) vs. end-diastolic (ED) aortic root measurements. In recent years, the trend has been towards end-diastolic aortic root measurements [11]. Yet, end-diastolic measurements are occasionally imprecise due to limited echocardiographic image quality. In particular, the insertion of the right coronary and non-coronary cusps is much better visible at mid-systole and facilitates reliable measurements. Given the fact that mid-systolic aortic root measurements correlate better with intraoperative measurements in the paediatric population, the 2010 ASE paediatric guidelines recommend aortic root measurements at mid-systole [17]. However, there are still several strong arguments to insist on end-diastolic aortic root measurements. First of all, end-diastolic aortic root measurements have a clear advantage of the well-defined time-point of measurement (i.e., ECG-triggered at the onset of the QRS complex) and the beneficial hemodynamic condition of stable aortic pressure. Furthermore, aortic valve competence is primarily maintained by diastolic AoAn and STJ shape. Therefore, the definition of end-diastolic aortic root diameters may be more relevant for clinical decisions, while trying to answer the question of the optimal AoAn diameter that is required to restore aortic valve. Due to these considerations, end-diastole has been recommended as the timepoint for echocardiographic aortic root measurement in adults by the ASE/EACVI [12]. Given this controversy, we decided to perform both end-diastolic and mid-systolic aortic root measurements in our current study.

### Rationale for matching of aortic root diameters

The variation of aortic root diameters found in the population-based HCHS study correlates well with the so far published studies of similar design [6–8, 18–25]. Previous studies revealed a marked heterogeneity of aortic root diameters that varied significantly depending on sex, age, and BSA.

Aortic root diameters were strongly related to male sex (+ 2.7 mm adjusted for BSA and age) in a study published by Devereux et al. [8]. The Framingham Heart Study showed on average 2.4 mm smaller aortic root dimensions in women than that of men of comparable age and BSA [7]. Similar sex effects have been demonstrated in a smaller Japanese population which revealed 3 mm larger absolute aortic root diameters in men vs. women [19]. A reverse trend after indexing the STJ and AscAo diameters for BSA has been reported in athletes and the general population [18, 25]. Similarly, we found significantly larger indexed aortic root diameters in women at all measured aortic root levels.

Previous echocardiographic studies revealed a constant association between aortic root diameters and age [18, 19, 24, 26]. All these studies described an increase of aortic root size by 0.7–1.0 mm/ decade in different populations. In the present study, we calculated the growth of aortic root diameters separately at all measured levels and found that AoAn grows slower (0.10 mm/decade) compared to the SoV (1.0 mm/decade), STJ (0.79 mm/decade), and AoAsc (0.71 mm/decade).

Strong correlation has been consistently reported between aortic root diameters and height, weight, and BSA in the normal adult population [7, 8, 18, 19, 24]. In line with previous studies, to these findings, we found no relevant difference between including height and weight vs. BSA alone in our multivariate linear regression model [8, 18]. Moreover, integrating more biological and clinical data did not improve the prediction of aortic root diameters. Hence, we created formulas for the calculation of individualized end-diastolic aortic root reference values that integrate age, sex, height, and weight as simple and readily available variables (Table 4). However, some limitations of such linear models should be considered. In particular, they are able to provide only limited information regarding the individual aortic root diameters, as they cover maximally 39% of the absolute variance (i.e., adjusted R-values from 0.19 to 0.39). This limitation should be considered in the clinical practice when defining “normal” aortic root diameters in individual patients.

### Limitations

Our study sample, which represents a sample of the first 10.000 subjects of the HCHS, originates from an at random selected population of the city of Hamburg. Although being a variegated, multicultural city, most subjects are of Caucasian ascend. Due to the exclusion of all subjects with relevant comorbidities, females were overrepresented in our sample. Furthermore, subjects included in HCHS were between 45 to 78 years old resulting in no representation of subjects at younger age. Therefore, the applicability of our findings in different populations should be done cautiously. Lastly, we combined the II and LL conventions in aortic root measurements which is not a validated approach. Further correlation studies comparing echocardiographic measurements with other imaging modalities (i.e., CMR, CT) are needed to establish a standardized and uniform measuring method.

## Conclusion

The present study provides reference values of aortic root diameters based on a standardized and validated echocardiographic protocol in a large, at random-selected population-based study. Furthermore, we defined age-, sex-, and BSA- adjusted aortic root diameters stratified by the time of heart cycle to support clinical decision-making algorithms in the monitoring and treatment of aortic valve and root disease.

## Supplementary Information

Below is the link to the electronic supplementary material.

Supplementary file1 (DOCX 652 kb)

Supplementary file2 (DOCX 16 kb)

## Data Availability

The data underlying this article cannot be shared publicly due to the privacy of individuals that participated in the study. The data will be shared on reasonable request to the corresponding author*.*

## Abbreviations

Asc: Ascending
Ao: Aorta/aortic
ASE: American Society of Echocardiography
BMI: Body mass index
BSA: Body surface area
EACVI: European Association of Cardiovascular Imaging
ED: End-diastolic/diastole
ESC: European Society of Cardiology
GFR: Glomerular filtration rate
HCHS: Hamburg City Health Study
hsCRP: High sensitivity C-reactive protein
IMT: Intima media thickness
IVST: Interventricular septum thickness
LAVI: Left atrial systolic volume indexed to body surface area
LVEDD: Left ventricular end-diastolic diameter
LVEDV: Left ventricular end-diastolic volume
LVEF: Left ventricular ejection fraction
MS: Mid-systolic/systole
NT-proBNP: N-terminal pro-B-type natriuretic peptide
NYHA: New York Heart Association
STJ: Sinotubular junction
TAPSE: Tricuspid annular peak systolic excursion

## Code availability

Will be shared on reasonable request.
