# Supplementary material for: Transthoracic echocardiographic reference values of the aortic root: results from the Hamburg City Health Study
Source: Int J Cardiovasc Imaging. 2021 Jul 29;37(12):3513–24. doi: 10.1007/s10554-021-02354-5 (PMC8604854; doi:10.1007/s10554-021-02354-5)
Supplement: Supplementary file 1 — Supplementary file1 (DOCX 652 kb) [file 10554_2021_2354_MOESM1_ESM.docx]

**12. Supplements**

**Tables**

**Table 5.** **End-diastolic aortic measurements indexed to BSA stratified by age and sex.**

|  |  |  | **Age categorized** | | | | | |  |
| --- | --- | --- | --- | --- | --- | --- | --- | --- | --- |
| ***End-diastolic diameters indexed to BSA (mm/m2)*** | | | **45-49** | **50-54** | **55-59** | **60-64** | **65-69** | **70+** | **p-value** |
| ***Male*** |  | | **(n= 96)** | **(n = 181)** | **(n = 123)** | **(n = 110)** | **(n = 94)** | **(n = 77)** |  |
|  | *Aortic annulus,*  *mm* | | 10.26  [10.04-10.48] | 10.61  [10.47-10.75] | 10.7  [10.51-10.88] | 10.62  [10.43-10.8] | 10.65  [10.46-10.85] | 10.81  [10.58-11.05] | 0.008 |
|  | *Sinus of Valsalva,*  *mm* | | 16.99  [16.62-17.35] | 17.34  [17.08-17.61] | 17.83  [17.51-18.15] | 18.49  [18.16-18.82] | 18.42  [18.01-18.83] | 18.85  [18.37-19.33] | <0.001 |
|  | *Sinotubular Junction, mm* | | 13.08  [12.75-13.41] | 13.7  [13.45-13.94] | 13.84  [13.6-14.08] | 14.37  [14.07-14.67] | 14.31  [14.01-14.6] | 14.29  [13.86-14.71] | <0.001 |
|  | *Ascending aorta,*  *mm* | | 14.71  [14.15-15.26] | 15.13  [14.74-15.51] | 15.04  [14.59-15.49] | 15.97  [15.53-16.41] | 15.74  [15.18-16.31] | 15.91  [15.36-16.46] | 0.001 |
| ***Female*** |  | | **(n = 128)** | **(n = 256)** | **(n = 248)** | **(n = 176)** | **(n = 109)** | **(n = 89)** |  |
|  | *Aortic annulus,*  *mm* | | 10.91  [10.73-11.08] | 11.07  [10.94-11.2] | 11.17  [11.02-11.31] | 11.19  [11.03-11.36] | 11.55  [11.34-11.76] | 11.52  [11.24-11.79] | <0.001 |
|  | *Sinus of Valsalva,*  *mm* | | 17.6  [17.3-17.9] | 17.81  [17.58-18.03] | 18.25  [18-18.51] | 18.53  [18.22-18.85] | 19.15  [18.8-19.5] | 19.16  [18.68-19.65] | <0.001 |
|  | *Sinotubular Junction, mm* | | 13.78  [13.5-14.06] | 13.92  [13.72-14.13] | 14.62  [14.39-14.84] | 14.61  [14.31-14.92] | 14.98  [14.65-15.31] | 15.06  [14.6-15.51] | <0.001 |
|  | *Ascending aorta,*  *mm* | | 15.82  [15.27-16.37] | 15.61  [15.28-15.94] | 16.14  [15.79-16.5] | 16.36  [15.88-16.84] | 17.12  [16.61-17.64] | 16.72  [16.12-17.32] | <0.001 |
| ***Overall*** |  | | **(n = 224)** | **(n = 437)** | **(n = 371)** | **(n= 286)** | **(n = 203)** | **(n= 166)** |  |
|  | *Aortic annulus,*  *mm* | | 10.63  [10.48-10.77] | 10.88  [10.78-10.97] | 11.01  [10.9-11.13] | 10.96  [10.84-11.09] | 11.15  [10.99-11.31] | 11.18  [10.99-11.37] | <0.001 |
|  | *Sinus of Valsalva,*  *mm* | | 17.34  [17.11-17.58] | 17.61  [17.43-17.78] | 18.11  [17.91-18.32] | 18.52  [18.29-18.75] | 18.83  [18.55-19.1] | 19.02  [18.68-19.36] | <0.001 |
|  | *Sinotubular Junction, mm* | | 13.48  [13.26-13.7] | 13.83  [13.67-13.98] | 14.36  [14.18-14.53] | 14.52  [14.3-14.74] | 14.69  [14.46-14.92] | 14.7  [14.39-15.02] | <0.001 |
|  | *Ascending aorta,*  *mm* | | 15.32  [14.92-15.72] | 15.39  [15.14-15.64] | 15.8  [15.51-16.08] | 16.2  [15.87-16.53] | 16.55  [16.15-16.95] | 16.31  [15.9-16.72] | <0.001 |

Values are presented as mean and 95% confidence interval. P-value for intergroup differences. Abbreviations as in Table 1.

**Table 6. Feasibility of aortic root measurements**

|  | **FEASIBILITY** | | | |  |
| --- | --- | --- | --- | --- | --- |
|  | Male | Female | Overall | p-value | |
|  | (n = 681) | (n = 1006) | (n = 1687) |  | |
| *ED Aortic annulus* | 601 (88.3) | 887 (88.2) | 1488 (88.2) | 1.000 | |
| *ED Sinus of Valsalva* | 659 (96.8) | 981 (97.5) | 1640 (97.2) | 0.446 | |
| *ED Sinotubular junction* | 576 (84.6) | 858 (85.3) | 1434 (85.0) | 0.742 | |
| *ED Ascending aorta* | 383 (56.2) | 538 (53.5) | 921 (54.6) | 0.286 | |
| *MS Aortic annulus* | 664 (97.5) | 983 (97.7) | 1647 (97.6) | 0.908 | |
| *MS Sinus of Valsalva* | 547 (80.3) | 836 (83.1) | 1383 (82.0) | 0.164 | |
| *MS Sinotubular junction* | 458 (67.3) | 691 (68.7) | 1149 (68.1) | 0.571 | |
| *MS Ascending aorta* | 358 (52.6) | 510 (50.7) | 868 (51.5) | 0.480 | |

**Table 7. Multiple linear regression analyses of absolute aortic root diameters (mm) measured in end-diastole with systolic or diastolic blood pressure as independent variables adjusted for age, sex, height, and weight.**

|  | **Systolic blood pressure model** | | | **Diastolic blood pressure model** | | |
| --- | --- | --- | --- | --- | --- | --- |
| **End-diastolic diameters** | R² | ß | p-value | R² | ß | p-value |
| **Aortic annulus** | 0.38 |  |  | 0.38 |  |  |
| *Age* |  | 0.010 | 0.793 |  | 0.01 | 0.793 |
| *Male sex* |  | 1.056 | <0.001 |  | 1.056 | 0.000 |
| *Height* |  | 0.028 | <0.001 |  | 0.028 | 0.000 |
| *Weight* |  | 0.028 | <0.001 |  | 0.028 | 0.000 |
| *Systolic blood pressure* |  | 0.000 | 1.000 |  |  |  |
| *Diastolic blood pressure* |  |  |  |  | 0.000 | 1.000 |
| **Sinus of Valsalva** | 0.41 |  |  | 0.42 |  |  |
| *Age* |  | 0.105 | <0.001 |  | 0.105 | 0.000 |
| *Male sex* |  | 2.213 | <0.001 |  | 2.213 | 0.000 |
| *Height* |  | 0.087 | <0.001 |  | 0.093 | 0.000 |
| *Weight* |  | 0.051 | <0.001 |  | 0.05 | 0.000 |
| *Systolic blood pressure* |  | 0.002 | 1.000 |  |  |  |
| *Diastolic blood pressure* |  |  |  |  | 0.002 | 1.000 |
| **Sinotubular Junction** | 0.32 |  |  | 0.32 |  |  |
| *Age* |  | 0.078 | <0.001 |  | 0.078 | 0.000 |
| *Male sex* |  | 1.104 | <0.001 |  | 1.104 | 0.000 |
| *Height* |  | 0.078 | <0.001 |  | 0.087 | 0.000 |
| *Weight* |  | 0.039 | <0.001 |  | 0.039 | 0.000 |
| *Systolic blood pressure* |  | 0.005 |  |  |  |  |
| *Diastolic blood pressure* |  |  |  |  | 0.005 | 1.000 |
| **Ascending aorta** | 0.19 |  |  | 0.19 |  |  |
| *Age* |  | 0.072 | 0.000 |  | 0.072 | 0.000 |
| *Male sex* |  | 1.223 | 0.012 |  | 1.223 | 0.012 |
| *Height* |  | 0.039 | 1.000 |  | 0.039 | 1.000 |
| *Weight* |  | 0.055 | <0.001 |  | 0.055 | 0.000 |
| *Systolic blood pressure* |  | 0.014 | 1.000 |  |  |  |
| *Diastolic blood pressure* |  |  |  |  | 0.014 | 1.000 |

**Supplemental Figures**

**Figure 6.** **Echocardiographic aortic root diameters at end-diastole and mid-systole in relation to age, sex, and body surface area.**


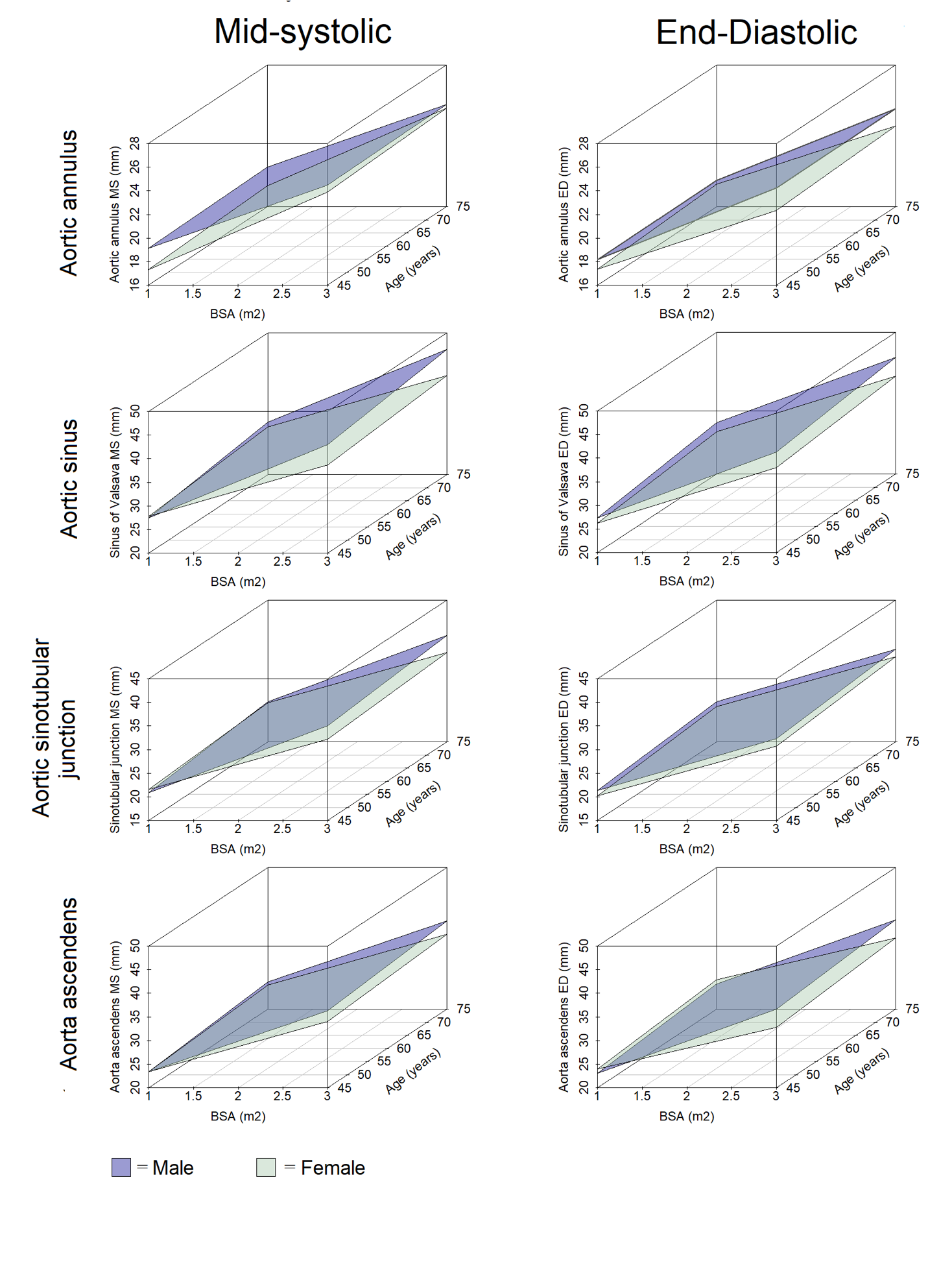


Surfaces represent the predicted mean aortic diameters end-diastolic and mid-systolic for males and females, respectively. *BSA = body surface area.*
