# Supplementary material for: Transthoracic echocardiographic reference values of the aortic root: results from the Hamburg City Health Study
Source: Int J Cardiovasc Imaging. 2021 Jul 29;37(12):3513–24. doi: 10.1007/s10554-021-02354-5 (PMC8604854; doi:10.1007/s10554-021-02354-5)
Supplement: Supplementary file 2 — Supplementary file2 (DOCX 16 kb) [file 10554_2021_2354_MOESM2_ESM.docx]

**Transthoracic echocardiographic reference values of the aortic root:**

**results from the Hamburg City Health Study**

**Appendix**

**List of used R packages**

extrafont_0.17   
fmsb_0.7.0
forcats_0.5.0
ggplot2_3.3.3    
ggpubr_0.4.0     
gridExtra_2.3   
kableExtra_1.3.1 
lme4_1.1-26   
magrittr_1.5
plot3d_1.3
psych_2.0.12     
purr_0.3.4
readr_1.4.0
reshape2_1.4.4
Rmisc_1.5   
Scatterplot3d_0.3-41
stringr_1.4.0
survival_3.2.7 
tableone_0.12.0   
tidyverse_1.3.0
